# Research Progress in the Cytogenetics of Sweetpotato and Its Wild Relatives

**DOI:** 10.3390/plants15172707

**Published:** 2026-09-03

**Authors:** Qiaoran Zhuansun, Yonghua Han

**Affiliations:** School of Life Sciences, Jiangsu Normal University, Xuzhou 221116, China; 18205008108@139.com

**Keywords:** sweetpotato, *Ipomoea*, wild relatives, cytogenetics, polyploidy, oligo-FISH

## Abstract

Cultivated sweetpotato (*Ipomoea batatas* (L.) Lam.), a hexaploid (2n = 6x = 90) crop, is the most economically important species within the morning glory genus *Ipomoea* (Convolvulaceae). Fourteen diploid *Ipomoea* species and several polyploid accessions have been confirmed to be closely related to sweetpotato, often termed its wild relatives. These wild species harbor abundant elite genes beneficial to sweetpotato improvement and thereby serve as indispensable germplasm reservoirs for breeding programs. In addition, several wild taxa are proposed as potential ancestors of domesticated sweetpotato. Nevertheless, the evolutionary origin and genomic architecture of cultivated sweetpotato have not yet been fully resolved. Cytological investigations, particularly chromosome karyotyping and meiotic pairing analyses, have been pivotal in unravelling the genomic architecture and evolutionary trajectories of polyploid taxa. Herein, we systematically summarize advances in chromosome counting, genome size, karyotyping, and meiotic pairing research on sweetpotato and its wild relatives.

## 1. Introduction

Cultivated sweetpotato (*Ipomoea batatas* (L.) Lam.) is a hexaploid (2n = 6x = 90) crop domesticated in the Americas and represents the most economically important species within the morning glory genus *Ipomoea* [1]. As the largest genus in Convolvulaceae, *Ipomoea* comprises approximately 800 species and is phylogenetically split into two major clades: the Old World Clade (OWC) and the New World Clade (NWC). Each clade is dominated by species endemic to the Old World and the New World, respectively. The New World taxa are further subdivided into five well-supported major clades, namely Clades A to E [2]. Fourteen diploid *Ipomoea* species (2n = 2x = 30), namely *I. australis*, *I. cordatotriloba*, *I. cynanchifolia*, *I. grandifolia*, *I. lactifera*, *I. lacunosa*, *I. leucantha*, *I. littoralis*, *I. ramosissima*, *I. splendor-sylvae*, *I. tenuissima*, *I. tiliacea*, *I. trifida*, and *I. triloba*, are the closest wild relatives of domesticated sweetpotato within *Ipomoea* [2,3]. Cultivated sweetpotato and its wild relatives fall into the A3 subclade of Clade A, a lineage widely known as the Batatas Clade [2]. In addition, several polyploid *Ipomoea* accessions (mostly tetraploids, with a few triploids and hexaploids) with uncertain identification are also closely related to sweetpotato [4]. These polyploid accessions have been variously identified as feral *I. batatas* [5,6] or *I. trifida* [7,8]. Several accessions were previously misidentified as *I. gracilis*, *I. littoralis*, and *I. tiliacea* [9]. Recently, Muñoz-Rodríguez et al. identified a tetraploid new species from these accessions, and named this new species *I. aequatoriensis* [10]. Another tetraploid taxon, *I. tabascana*, has long been hypothesized to be either an interspecific hybrid between sweetpotato and diploid *I. trifida* [10,11], or a potential tetraploid progenitor of cultivated sweetpotato [12].

**Figure 1:**
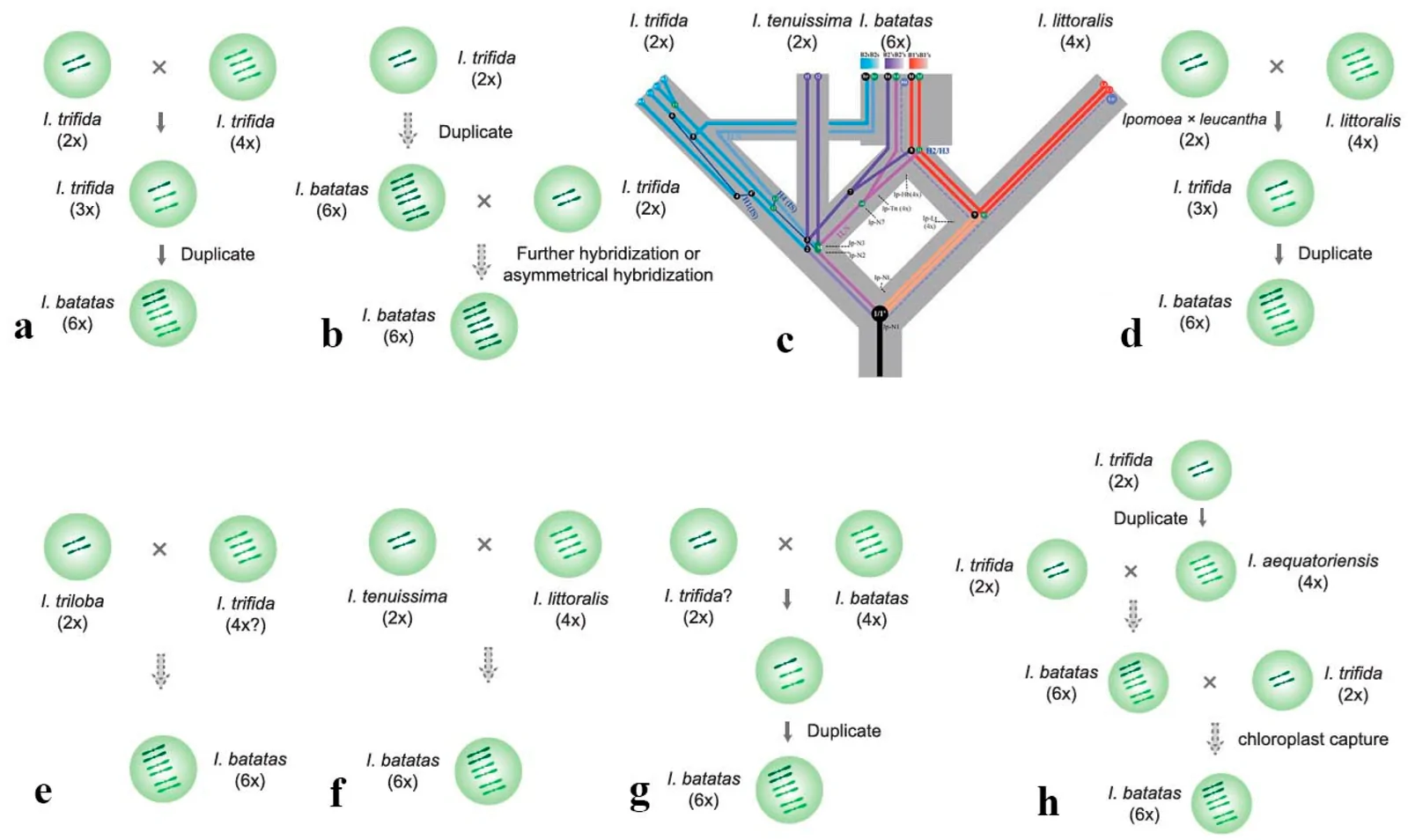
Hypotheses to describe the origin of cultivated sweetpotato (reproduced from Yan et al. [13]). (**a**) The autopolyploid hypothesis suggests that *I. trifida* is the only progenitor of sweetpotato [14]. (**b**) Sweetpotato originated from *I. trifida*, and after the speciation of sweetpotato, a hybridization event occurred between sweetpotato and *I. trifida* [3]. (**c**) The species tree embodying the two hybridization networks demonstrates the segmental allopolyploid hypothesis [15]. (**d**) The allopolyploid hypothesis of Nishiyama [16]: sweetpotato is derived from a triploid *I. trifida* that arose from hybridization of *Ipomoea × leucantha* and *I. littoralis*. (**e**) The allopolyploid hypothesis of Austin [17]: sweetpotato arose via hybridization between *I. trifida* and *I. triloba*. (**f**) The allopolyploid hypothesis of Gao et al. [12]: sweetpotato arose via hybridization between *I. tenuissima* and *I. littoralis*. (**g**) The allopolyploid hypothesis of Yang et al. [18] and Yan et al. [1]: sweetpotato is likely to be derived from a triploid that arose from a cross between *I. trifida* and *I. batatas* 4x. (**h**) The allopolyploid hypothesis of Muñoz-Rodríguez et al. [10]: *I. aequatoriensis* arose from a whole-genome duplication in *I. trifida*. Sweetpotato (*I. batatas*) is likely to be derived from a cross between *I. trifida* and *I. aequatoriensis*. Subsequent introgression between *I. trifida* and sweetpotato resulted in chloroplast capture from *I. trifida*.

Most of the aforementioned species have long been hypothesized as putative progenitors of cultivated sweetpotato. However, the origin and genome architecture of sweetpotato remain controversial. Three polyploidization hypotheses, namely autopolyploidy, allopolyploidy, and segmental allopolyploidy, have been put forward based on various studies [13]. The autopolyploid hypothesis posits that sweetpotato is an autopolyploid and that *I. trifida* is its only wild ancestor based on cytogenetic analysis [14] (Figure 1a) and phylogenetic analysis [3] (Figure 1b). The second origin hypothesis suggests that sweetpotato is a segmental allopolyploid that carries three partially differentiated subgenomes originally from *I. trifida*, *I. tenuissima*, and *I. littoralis*, based on an analysis of conserved single-copy genes [15] (Figure 1c). The third allopolyploid hypothesis suggests that sweetpotato was formed by hybridization between two progenitor species but that the identities of the progenitor species are controversial based on cytogenetic analysis [16] (Figure 1d), morphological data [17] (Figure 1e), an analysis of nucleotide sequence variation in the *Waxy* gene intron [12] (Figure 1f), phylogenetic analyses of homologous haplotypes [1,18] (Figure 1g), and morphological and phylogenetic analyses [10] (Figure 1h). Recently, a phased chromosome-level genome assembly of hexaploid sweetpotato has provided new insights into sweetpotato origin by analyzing sequence similarities and phylogenetic relationships between sweetpotato haplotypes and its wild relatives [19]. The study suggests that *I. aequatoriensis* may be a direct descendant of one progenitor, while the other donor(s) remain unknown, and *I. trifida* may not be the direct progenitor of hexaploid sweetpotato. It further reveals that the sequences contributed by different wild species are not distributed in typical subgenomes but are intertwined along chromosomes. This pattern may result from the known autopolyploid-like random pairing among homoeologous chromosomes during meiosis in hexaploid sweetpotato [20], which allows the exchange and replacement of sequences initially contributed by different donors. Accordingly, the authors propose that sweetpotato can be tentatively classified as a segmental allopolyploid.

Cytogenetic analyses, particularly karyotyping and observations of meiotic chromosome pairing, are widely applied to elucidate interspecific relationships and the origin of polyploid species. Early cytogenetic studies on sweetpotato and its wild relatives were mostly restricted to simple chromosome counting. Owing to their small, morphologically uniform chromosomes, precise discrimination of homologous chromosomes and high-resolution karyotyping have long remained unachievable [21,22,23,24]. Rapid advances in molecular cytogenetic techniques have greatly promoted cytogenetic investigations of sweetpotato and its wild relatives. In particular, fluorescence in situ hybridization (FISH) based on pooled single-copy oligonucleotide (oligo) probes (oligo-FISH) enables the accurate visualization and unambiguous identification of individual chromosomes, resolving the long-standing challenge of indistinguishable chromosomes [25,26]. This technique facilitates high-resolution karyotyping, subgenome composition analysis, and dynamic tracking of meiotic chromosome behavior. Herein, we systematically review advances in cytogenetic research of sweetpotato and its wild relatives.

## 2. Chromosome Number of Sweetpotato and Its Wild Relatives

Chromosome number constitutes a core cytogenetic trait for species delimitation. *Ipomoea* species exhibit ploidy levels of 2x, 4x, and 6x, with a basic chromosome number of *x* = 15 [27,28]. The definitive chromosome number of sweetpotato was not confirmed until 1953 [29]. As early as 1929, Kano preliminarily estimated the somatic chromosome number of sweetpotato to be roughly 42 [30]. In 1937, King and Bamford performed cytogenetic investigations across the Convolvulaceae family and found that the somatic chromosome numbers of most sampled species were multiples of the base chromosome number x = 15. They observed more than 60 chromosomes in sweetpotato somatic cells and thereby proposed that *I. batatas* was a hexaploid with roughly 90 somatic chromosomes [31]. Subsequent meiotic analyses by Ting and Kehr determined the gametic chromosome number to be 45, providing definitive cytogenetic evidence that sweetpotato has 90 somatic chromosomes [29]. Although cultivated sweetpotato typically maintains a stable somatic chromosome complement of 2n = 6x = 90, aneuploid individuals have been frequently observed across various cultivars [32,33,34].

Among sweetpotato relatives, Wolcott first reported a chromosome count of 2n = 2x = 30 for *I. lacunose* [35], which was later confirmed by Jones [27]. The same number was subsequently recorded for *I. triloba*, *I. tiliacea*, and *I. ramoni* (=*I. trifida*) [27,36], while counts for additional wild relatives were later determined by Jarret et al. [37] and Sun et al. [4].

## 3. Genome Size of Sweetpotato and Its Wild Relatives

Due to their high basic chromosome number and minute chromosomes, species within the genus *Ipomoea* pose substantial challenges to conventional cytological observation. Genome size, defined as the 2C DNA content of non-replicated somatic nuclei, serves as a robust quantitative parameter in modern cytogenetics [38]. Speciation is frequently accompanied by gains and losses of genomic DNA. Comparisons of genome size therefore serve as a powerful tool for unravelling phylogenetic relationships and clarifying the systematics of closely related taxa [39,40]. Within a taxon or complex, genome size is also a first indicator of ploidy level and hybridisation events [41,42]. Nuclear DNA content can be rapidly and reliably quantified via flow cytometry using DNA-specific fluorochromes [43].

Arumuganathan and Earle first quantified the 2C value of *I. batatas* at 3.31 pg using flow cytometry [44], followed by Marie and Brown, who reported a 2C value of 3.51 pg [45]. Ozias-Akins and Jarret enlarged the flow cytometric analysis to 42 accessions of sweetpotato and its wild relatives [46] (Table 1). In a more recent investigation, Srisuwan et al. carried out identical flow cytometric quantification across 22 accessions of sweetpotato and its wild relatives [47] (Table 1). Despite the systematic difference in 2C values from the two studies, nuclear DNA content showed a significant positive correlation with ploidy level across all tested species. This correlation was particularly prominent within single species harbouring multiple cytotypes. This result demonstrates that ploidy levels can be reliably inferred from genome size data across polyploid series. For instance, Ozias-Akins and Jarret identified two Ecuadorian *I. batatas* accessions as tetraploid [46], corroborating earlier cytological evidence from Jarret et al., who confirmed the tetraploid identity of these feral sweetpotato lines through mitotic chromosome counting of root-tip squashes [37]. Two genotypes identified as *I. batatas* var. *apiculata* also displayed 2C values characteristic of tetraploid sweetpotato [46]. Furthermore, this study documented tetraploid cytotypes of *I. cordatotriloba* for the first time. Mitotic root-tip chromosome counts for one *I. cordatotriloba* accession revealed 2n = 4x = 60, which was fully consistent with ploidy estimates obtained via flow cytometry.

Regarding the evolutionary origin of cultivated hexaploid sweetpotato, Kobayashi hypothesized that this hexaploid species arose directly via fusion of unreduced (2n) gametes from diploid and tetraploid *I. trifida* [49]. Based on nuclear DNA content analyses, Srisuwan et al. proposed alternative or complementary hypotheses distinct from Kobayashi’s single-step origin hypothesis [48]. They proposed that hexaploid *I. batatas* originated from *I. trifida* through a triploid-bridge pathway. Two routes may generate the required triploid intermediate. First, pollination of a tetraploid individual with normal haploid (n) pollen from a diploid parent yields a triploid hybrid. This triploid can undergo somatic chromosome doubling to generate fertile hexaploid progeny. Second, triploids can also be produced by intercrossing two diploid plants, where one parent generates unreduced gametes [48].

## 4. Karyotype Analysis of Sweetpotato and Its Wild Relatives

### 4.1. Karyotype Analysis Based on Conventional Aceto-Squash Staining

**Figure 2:**
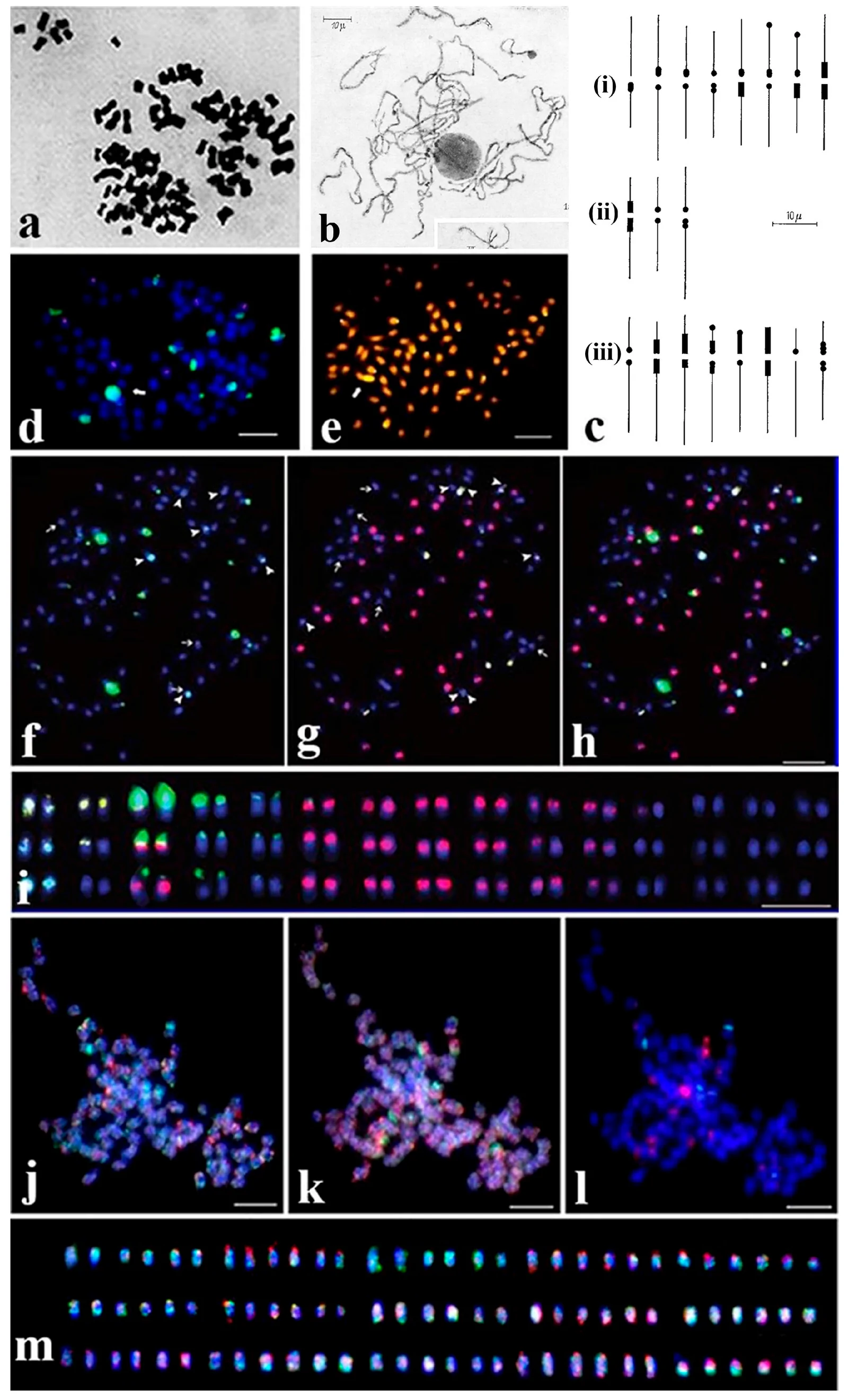
Karyotype analysis and chromosome identification in *I. batatas*. (**a**) Somatic metaphase chromosomes from Sinha and Sharma [23]. (**b**) Pachytene nucleus showing differentiated chromosomes from Magoon et al. [51]. (**c**) Ideogram at pachytene showing the 19 chromosomal types from Magoon et al. [51]. (**i**) Eight metacentric chromosome types, with arm ratios of 1:1.3 or lower; (**ii**) Three submetacentric chromosome types, with arm ratios ranging from 1.4 to 2.0; (**iii**) Eight acrocentric chromosome types, with arm ratios of 2.1 or higher. (**d**) One accession of hexaploid *I. batatas* showing 12 signals of 18S, and six 5S signals from Srisuwan et al. [11]. (**e**) CMA staining in *I. batatas*. The arrows indicate the biggest chromosome with a strong CMA^+^ band from Srisuwan et al. [11]. (**f**) FISH with the probe cocktail of 35S and 5S in *I. batatas* from Sun et al. [47]. (**g**) The same spread in (**f**) was reprobed with *Itf_1* and *Itf_2*. (**h**) The composite picture including four repetitive sequences. (**i**) The chromosomes are extracted from (**h**). (**j**) Simultaneous hybridization of oligo probes L1 (green) and L2 (red) on mitotic metaphase cell from Sun et al. [4]. (**k**) Simultaneous hybridization of oligo probes L3 (green), L4 (red), 15–1 (green), and 15–2 (red) on same cell in (**j**). (**l**) The same cell in (**j**) was reprobed with 5S (green) and 35S (red) rDNA probes. (**m**) The chromosomes with the signals of oligo probes digitally excised from the same cells in (**j**) and (**k**), respectively.

Karyotype analysis is the basic content of cytogenetic research, which can offer vital clues to delimit species relationships and infer polyploid origins [50]. Nevertheless, comprehensive karyological datasets for sweetpotato and its wild relatives remain extremely limited. Conventional aceto-squash staining techniques were initially utilized to analyze the karyotypes of sweetpotato and its two diploid relatives *I. lacunosa* and *I. triloba* [21,22,23,24]. These studies revealed that the chromosomes of these species are generally small and morphologically similar (Figure 2a). This morphological similarity impedes both accurate karyotyping and phylogenetic resolution among the studied taxa.

Notably, the minute chromosome size turns out to be a definite advantage for pachytene karyological analysis. Unlike mitotic metaphase chromosomes, well-differentiated pachytene chromosomes exhibit abundant diagnostic morphological markers that enable reliable chromosome identification. Using suitable traits, such as arm ratio, chromomeric pattern, nucleolar association, etc., Magoon et al. successfully distinguished 40 out of the 45 haploid pachytene chromosomes and classified them into 19 distinct chromosomal types [51] (Figure 2b,c). Among these 19 types, eight types were each represented by a single chromosome, six types by two chromosomes, two types by three chromosomes, two types by four chromosomes, and one type by six chromosomes. This observation can be interpreted as follows: (i) the eight chromosomal types present singly in the haploid complement are not represented in all three genomes; (ii) the six types occurring in duplicate are common to two of the three genomes; (iii) the two types represented in triplicate are present in all three genomes. Accordingly, pachytene cytological observation demonstrates that the ancestral subgenomes of sweetpotato can be discriminated based on one or more of the eight chromosomal types. Such cytogenetic evidence rules out an autopolyploid origin and substantiates an allopolyploid origin from related ancestors.

### 4.2. Karyotype Analysis Based on FISH, and Fluorochrome Banding

Enzymatic digestion coupled with hypotonic treatment substantially improves chromosome spreading quality, and these optimized protocols have greatly facilitated the application of chromosome banding and FISH for plant karyotyping and chromosome identification. FISH is a robust tool for chromosome discrimination, as its signals can serve as diagnostic cytological landmarks [52]. Among the various probe types, tandem repetitive DNA sequences are the most widely used FISH probes for plant chromosome identification; these sequences localize to discrete chromosomal domains and are typically classified as centromeric, subtelomeric, or intercalary repeats. However, it often takes a significant effort to develop such probes or probe sets to identify all chromosomes in each species. Moreover, because repeat-based probes target only specific chromosomal regions, they may fail to reliably identify corresponding chromosomes across different genotypes or to recognize homeologous chromosomes among related species. Furthermore, aside from the conserved 5S and 35S (18S–5.8S–26S) ribosomal DNA (rDNA) repeats, species-specific repeat probes remain unavailable for most plant species [52,53].

In pioneering FISH-based cytogenetic research on sweetpotato and its wild relatives, Srisuwan et al. characterized the distribution and organization of 5S and 18S rDNA across ten *I. batatas* cultivars, five tetraploid *I. trifida* accessions, and six related species (five diploids: *I. trifida*, *I. triloba*, *I. tiliacea*, *I. leucantha*, *I. setosa*; one tetraploid: *I. tabascana*) [11] (Table 1). All test materials were also analyzed via fluorochrome banding (GC-specific staining with chromomycinA3, CMA) (Table 1). Combining the chromosomal distribution of 18S and 5S rDNA loci with CMA^+^ banding patterns allowed for higher-resolution karyotype delineation (Figure 2d,e). Based on chromosomal morphological characteristics, tetraploid *I. trifida* exhibits a closer evolutionary relationship with sweetpotato relative to *I. tabascana*. Collectively, these cytogenetic data support *I. trifida* as the putative progenitor of *I. batatas*, whereas *I. tabascana* likely originated from hybridization between hexaploid sweetpotato and diploid *I. trifida*.

Cytological analyses of a single sweetpotato cultivar were additionally performed using silver nitrate staining, 35S rDNA-FISH, and self-genomic in situ hybridization (self-GISH) as documented in Tang et al. [54]. Distinct numbers of silver-stained nucleolar organizer regions (Ag-NORs), ranging from 12, 16 to 18 labeled sites, were detected within the interphase nuclei of the examined cultivar. Consistently, 35S rDNA-FISH assays revealed 16 to 18 hybridization signals exhibiting variable fluorescence brightness. Meanwhile, self-GISH yielded intense fluorescent signals uniformly covering all mitotic chromosomes. Subsequent to this investigation, An et al. expanded the karyotype characterization of the same cultivar via combined 4′,6-diamidino-2-phenylindole (DAPI) banding and 5S rDNA-FISH assays [55]. DAPI banding established the karyotype formula as 2n = 6x = 90 = 72m + 18sm (18SAT), with satellites located on chromosomes 1, 3, and 6. FISH mapping of 5S rDNA loci further resolved three clearly distinguishable pairs of hybridization signals across the chromosome complement, providing additional cytogenetic markers for chromosome identification.

Comparative karyotyping of sweetpotato and five wild relatives utilizing two satellite repeats (*Itf_1*, *Itf_2*) from the diploid *I. trifida* genome used as FISH probes was performed by Sun et al. [47] (Figure 2f–i) (Table 1). The similarity in the pattern of signal distribution on chromosomes was assessed. Their results revealed a similar karyotype between *I. tenuissima* and *I. × leucantha*, suggesting a close relationship between them. Notably, the combined signal pattern of *Itf_1* and 35S rDNA was detected exclusively in *I. trifida*, *I. tabascana*, and *I. batatas*, pointing to their closer affinity. Meanwhile, chromosomes carrying 5S rDNA appeared conserved across all six species, as they consistently co-localized with *Itf_2* signals, yielding a shared distribution pattern. Collectively, these FISH data provide pivotal insights into the interspecific evolutionary relationships among the species examined.

### 4.3. Karyotype Analysis Based on Oligo-FISH

In all karyotypes of sweetpotato and its wild relatives described above, reliable discrimination of all chromosomes within one cell remains unattainable due to the scarcity of robust cytogenetic markers. Oligo-FISH represents a powerful strategy for chromosome discrimination and karyotyping [25,26]. Furthermore, since the single-copy sequences exploited for oligo probe design are predominantly derived from conserved gene-coding regions, oligo probes designed for one species can be used in other plants related to the target species. This property endows oligo probes with a distinct advantage for comparative karyotype analysis among related species. Two classes of oligo-based probe systems have been established for plant karyotyping. The first class consists of oligo pools targeting either discrete chromosomal segments or whole individual chromosomes. These probes produce clear chromosome-painting signals, and each painting probe enables unambiguous identification of one single chromosome. The second probe class comprises oligo sets derived from multiple regions of multiple chromosomes. These probes generate unique barcode-like signal patterns on different chromosomes, and a single probe pool can simultaneously distinguish multiple chromosomes. To date, oligo-based probes have been widely applied to karyotyping numerous plant genera [26,56].

Four oligo-FISH barcode probes and two chromosome painting probes developed from model diploid *Ipomoea nil* were deployed to fully discriminate all chromosomes and map rDNA loci across sweetpotato and ploidy-diverse wild relatives for the first time [4] (Table 1). Comparative karyotyping revealed that *I. trifida* shared the greatest karyotypic resemblance to sweetpotato among the 12 investigated diploid species (two taxa, *I. australis* and *I. lactifera*, were unavailable for analysis). Furthermore, of these 12 diploids, only *I. trifida* possessed rDNA locus arrangements fully congruent with those observed in sweetpotato and other polyploid relatives. These findings indicate that *I. trifida* is the closest diploid relative of sweetpotato, whereas all other diploid taxa can be ruled out as candidate diploid ancestors. The study also revealed that *I. aequatoriensis* and tetraploid *I. batatas* possessed identical rDNA distribution profiles and highly congruent karyotypes, rendering the two taxa cytogenetically indistinguishable. By contrast, *I. tabascana* was karyotypically well-differentiated from both *I. aequatoriensis* and tetraploid *I. batatas*. Notably, *I. tabascana* exhibited divergent 35S rDNA signal intensities across its four homologous chromosomes: two chromosomes harbored intense hybridization signals, whereas the other two carried faint signals. This pattern segregates the four homologous chromosomes into two distinct pairs, furnishing additional cytogenetic evidence that *I. tabascana* most likely arose via genome doubling of structurally homologous, closely related subgenomes, rather than through interspecific hybridization between sweetpotato and *I. trifida* [10,11]. Consistent evolutionary inferences were also reached by Yan et al., who performed phylogenetic and clustering analyses on simulated tetraploid hybrids of *I. trifida* and sweetpotato [1]. Likewise, cultivated sweetpotato and hexaploid *I. trifida* exhibited a similar karyotype and the same distribution of 5S and 35S rDNA. Nevertheless, both taxa exhibited inter-homologue divergence in chromosome length, morphology, and rDNA signal intensity, with such variation far more pronounced in sweetpotato (Figure 2j–m), consistent with its proposed segmental allopolyploid ancestry [8,15].

## 5. Meiotic Chromosome Pairing and Genomic Architecture Analyses of Sweetpotato and Its Polyploid Wild Relatives

Chromosome pairing analyses are widely adopted to infer the genomic constitution of polyploids, as autopolyploids are theoretically predicted to form abundant multivalents during meiosis [57,58]. Nonetheless, evaluations relying solely on meiotic pairing patterns have notable limitations. Over evolutionary time, autopolyploids may establish stable meiotic behavior where homologous chromosomes predominantly synapse as bivalents instead of multivalents [59,60,61]. Furthermore, tracking the pairing behavior of individual chromosomes and accurately discriminating diverse multivalent configurations across successive meiotic stages pose substantial technical obstacles in polyploid taxa. These methodological drawbacks have fueled longstanding contradictory interpretations regarding the genome structure of sweetpotato, giving rise to competing autopolyploid and allopolyploid hexaploid models from early cytogenetic investigations [29,51,62].

**Figure 3:**
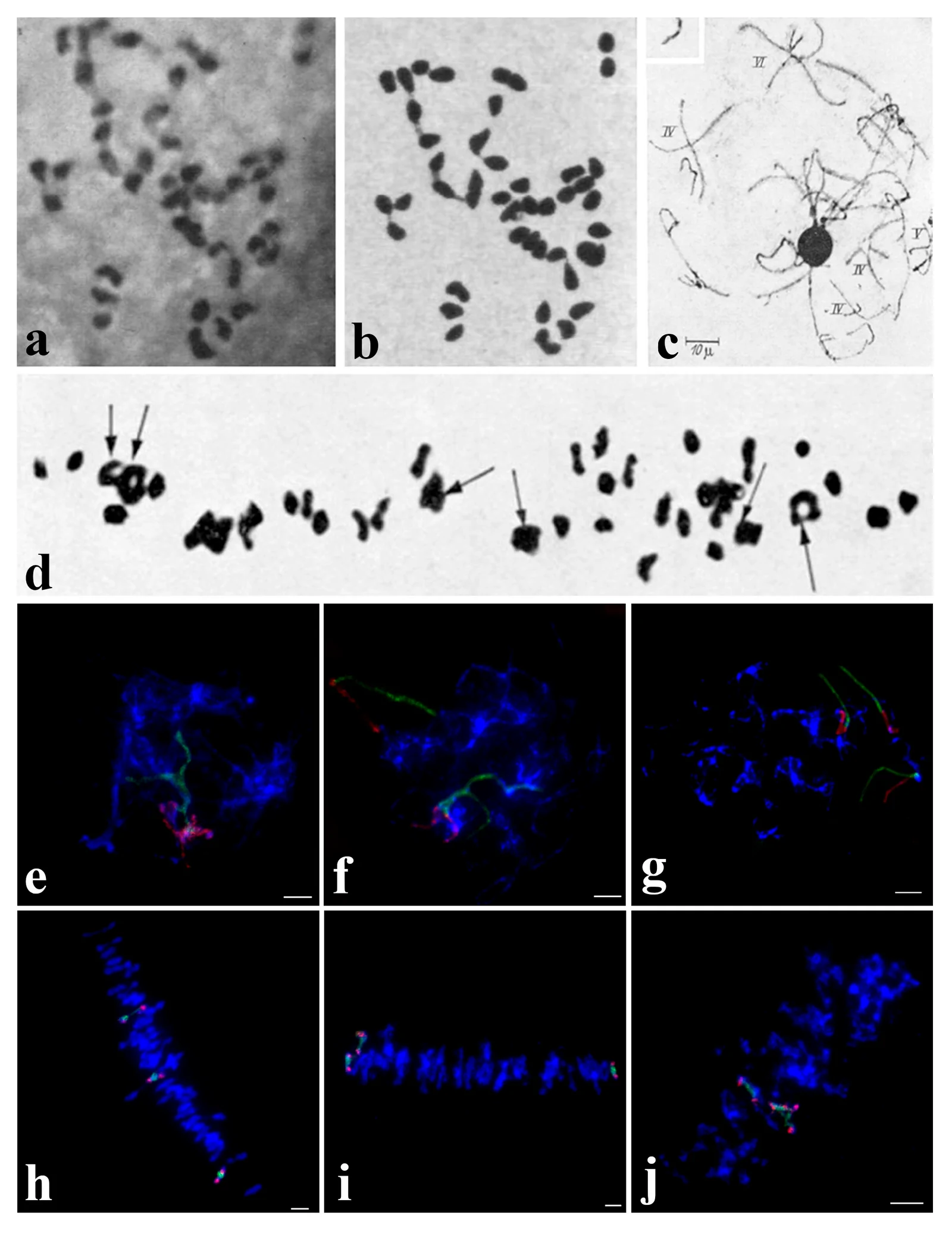
Meiotic chromosome pairing in *I. batatas*. (**a**) Photomicrograph of chromosomes of one sweetpotato variety at metaphase I, showing secondary association of 3 groups of three bivalents and 7 groups of two bivalents from Ting and Kehr [29]. (**b**) A drawing of the 45 bivalents from the same cell as in (**a**). Here some associated bivalents are connected by strands of chromatin material. (**c**) Pachytene-stage multivalent chromosome associations reported by Magoon et al. [51], showing one cell with 3 quadrivalents (IV), 1 pentavalent (V), and 1 hexavalent (VI). (**d**) Quadrivalent chromosomal associations (arrows) at metaphase I from Magoon et al. [51]. (**e**–**j**) Chromosome painting in zygotene–pachytene (**e**–**g**) and metaphase I cells (**h**–**j**) from Sun et al. [34]. The six chromosomes painted by oligo probes paired as a hexavalent (**e**,**h**), one bivalent and one quadrivalent (**f**,**i**), and three independent bivalents (**g**,**j**).

The first meiotic cytological investigation of sweetpotato was conducted in two varieties. A full set of 45 bivalents was clearly discernible in both accessions, and no multivalent configurations were detected across all observed meiotic cells [29]. Notably, consistent secondary associations of two to three bivalents were universally observed at metaphase I (Figure 3a,b). Ting et al. further characterized meiotic behaviors in eight commercial sweetpotato varieties and confirmed the ubiquitous occurrence of bivalent secondary associations at metaphase I across all tested materials [63].

As first described by Lawrence [64], secondary bivalent association occurs when chromosomes carry small homologous segments insufficient to form stable chiasmata, yet maintain weak interchromosomal homology that drives their aggregation at meiosis. Such secondary pairing is broadly accepted as robust cytogenetic evidence for partially homologous chromosome sets within allopolyploid taxa. Integrating the consistent occurrence of secondary bivalent associations and the complete absence of meiotic multivalents, Ting and Kehr proposed that sweetpotato originated from interspecific hybridization between two genetically divergent yet closely related progenitors: a tetraploid species (2n = 4x = 60) and a diploid species (2n = 2x = 30). This sterile hybrid subsequently regained fertility via spontaneous natural chromosome doubling [29].

Nevertheless, Magoon et al. reported that multivalent associations were very common, in addition to the predominance of bivalent chromosomal configurations, at both the pachytene and metaphase I stages [51] (Figure 3c,d). The occurrence of such multivalents indicates extensive chromosomal homology among the constituent genomes of sweetpotato. Multivalents were frequently quadrivalents and hexavalents, while trivalent and pentavalent associations were relatively rare. The hexavalents at pachytene were resolved into three distinct types based on their morphology. The first type consisted of homomorphic, acrocentric chromosomes that were evidently present in all three genomes. In the second type, chromosomes of only two of the genomes were homomorphic and metacentric, while that from the third genome was acrocentric, probably reflecting the chromosomal differentiation that had occurred in the latter genome. In the third hexavalent type, where all the participating chromosomes were metacentric, only two were similar in their chromomeric pattern. Among quadrivalent associations, three distinct types could be distinguished based on the morphology of their constituent chromosomes. All three types involved homologous chromosomes, and the specific types of the participating chromosomes were also determined. Thus, the frequency of quadrivalent association, together with the occurrence of six chromosomal types in duplicate, suggests a closer homology between two of the three parental genomes. The third genome nevertheless shows some homology to the other two, as evidenced by the occurrence of three distinct hexavalent configurations. Based on these cytological observations, the authors inferred that sweetpotato possesses three partially homologous ancestral subgenomes, wherein two subgenomes share higher sequence homology with each other than with the third. This cytogenetic model was later interpreted by Oracion et al. as the segmental allopolyploid (or segmental auto-allopolyploid) hypothesis [8].

Consistent with the aforementioned model of segmental allopolyploid constitution, the genomic formula B_1_B_1_B_2_B_2_B_2_B_2_ was proposed for sweetpotato based on meiotic cytological analyses of synthetic hexaploids derived from diploid and tetraploid *I. trifida* and also their F_1_ hybrids with sweetpotato. The B_1_B_1_ subgenome corresponds to diploid *I. trifida*, while the B_2_B_2_B_2_B_2_ component derives from tetraploid *I. trifida* [9,14]. Furthermore, from their subsequent meiotic pairing study of inter- and intraploidy hybrids within the *I. trifida* complex, Shiotani and Kawase concluded that genomes B_1_ and B_2_ are homologous and that sweetpotato and polyploid *I. trifida* are therefore autopolyploids, consisting of the B genome of diploid *I. trifida* [62].

Oligo-based chromosome painting probes targeting two chromosomes of *I. nil* have recently been developed and applied to dissect meiotic pairing patterns in *I. tabascana*, *I. batatas*, and hexaploid *I. trifida* [34]. Chromosome painting detected quadrivalents in up to 82% of zygotene–pachytene cells of *I. tabascana*, indicative of strong homology across four homologous chromosomes. In addition, the four homologous chromosomes painted by probes from a single chromosome could be resolved into two distinct pairs based on the presence or absence of satellites and secondary constrictions. Therefore, it is reasonable to assume that *I. tabascana* is an autotetraploid, likely derived from genome doubling of structurally similar homologous genomes, rather than a hybrid between *I. batatas* and diploid *I. trifida*. In hexaploid *I. trifida* and *I. batatas*, the six homologous chromosomes predominantly paired as a single hexavalent at zygotene–pachytene, suggesting considerable homology among the three subgenomes. The concurrent high frequency of bivalent and tetravalent pairing at zygotene–pachytene and metaphase I further indicated closer homology between two of the three subgenomes (Figure 3e–j). However, a marked difference in pairing behavior was evident between the two species: the proportion of prophase I and metaphase I cells displaying configurations involving all homologous chromosomes was substantially higher in *I. trifid* than in *I. batatas*, reflecting greater subgenomic similarity in the former. Thus, these data tend to support an autohexaploid origin for *I. trifida*, whereas sweetpotato is more likely a segmental allohexaploid.

## 6. Conclusions

Modern molecular cytogenetic techniques, especially oligo-based FISH, have overcome bottlenecks in conventional cytogenetic studies and greatly advanced cytogenetic investigations of sweetpotato and its wild relatives.

Regarding the origin of sweetpotato, seven out of the eight hypotheses proposed to date posit the involvement of *I. trifida* (Figure 1). Comparative karyotyping based on oligo-FISH demonstrated that *I. trifida* exhibits the highest karyotypic similarity to sweetpotato among the surveyed diploid species. Furthermore, only *I. trifida* shows rDNA locus arrangements fully congruent with those observed in sweetpotato and its polyploid relatives such as *I. aequatoriensis*, tetraploid *I. batatas*, and hexaploid *I. trifida*. These results identify *I. trifida* as the closest diploid relative of sweetpotato. Nevertheless, the most recent study based on phased chromosome-level genome assemblies of hexaploid sweetpotato suggests that *I. trifida* may not be the direct progenitor of hexaploid sweetpotato, and *I. aequatoriensis* could be a direct descendant of one progenitor, while the other donor(s) remain unknown.

For the genome architecture of sweetpotato, among the three proposed polyploidization hypotheses, the segmental allopolyploid hypothesis has received the strongest support from cytogenetic analyses. These cytogenetic observations are consistent with genome-based inferences, which tentatively categorize sweetpotato as a segmental allopolyploid on the basis of phased chromosome-level genome assemblies of hexaploid sweetpotato.

## Figures and Tables

**Table 1:** Summary of 2n/x, number of 5S/35S rDNA sites, CMA-positive bands, and 2C nuclear DNA content.

Species	2n (x = 15) ^a^	Number of 5S rDNA ^b^	Number of 18S or 35S rDNA ^c^	Number of CMA^+^ Bands ^d^	DNA Content (pg/2C) ^e^
*I. australis*	–	–	–	–	–
*I. cordatotriloba*	2n = 2x = 30	2/–/–	8/–/–	–	1.6–1.8/–
*I. cynanchifolia*	2n = 2x = 30	2/–/–	10/–/–	–	1.7/–
*I. grandifolia*	2n = 2x = 30	2/–/–	10/–/–	–	–
*I. lactifera*	–	–	–	–	–
*I. lacunosa*	2n = 2x = 30	2/–/–	6/–/–	–	1.5–1.8/–
*I. leucantha*	2n = 2x = 30	2/2/2	10/8/8	8	1.6/1.06
*I. littoralis*	2n = 2x = 30	2/–/–	4/–/–	–	2.4/–
*I. ramosissima*	2n = 2x = 30	2/–/–	14/–/–	–	1.7/–
*I. splendor–sylvae*	2n = 2x = 30	2/–/2	4/–/4	–	–
*I. tenuissima*	2n = 2x = 30	2/–/2	8/–/10	–	1.8/–
*I. tiliacea*	2n = 2x = 30	2/2/–	8/8/–	8	–/1.12
*I. trifida*	2n = 2x = 30	2/2/2	6/6/6	6	1.7/1.11
*I. triloba*	2n = 2x = 30	2/2/–	8/6/–	6	1.5–1.7/1.01
*I. aequatoriensis*	2n = 4x = 60	4/–/–	12/–/–	–	–
*I. batatas 4x (or I. trifida 4x)*	2n = 4x = 60	4/4/–	12/10/–	12	3.0–3.6/2.07
*I. tabascana*	2n = 4x = 60	4/4/4	12/10/14	10	2.6/2.03
*I. batatas*	2n = 6x = 90	6/6/6	17/12,16 or18/18	12,16 or18	4.6–5.2/3.22
*I. trifida* 6x	2n = 6x = 90	6/–/–	17/–/–	–	–

^a^ Chromosome number from Sun et al. [4] and Jarret et al. [37]. ^b^ Number of 5S rDNA sites, from left to right, are cited from Sun et al. [4], Srisuwan et al. [11], and Sun et al. [47], respectively. ^c^ Number of 35S rDNA sites, from left to right, are cited from Sun et al. [4], Srisuwan et al. [11], and Sun et al. [47], respectively. ^d^ Numbers of CMA^+^ bands are cited from Srisuwan et al. [11]. ^e^ DNA content values are cited from Ozias-Akins and Jarret [46] and Srisuwan et al. [48], respectively.

## Data Availability

No new data were created or analyzed in this study. Data sharing is not applicable to this article.
